# Supplementary material for: Genome report: First reference genome of Vaccinium floribundum Kunth, an emblematic Andean species
Source: G3 (Bethesda). 2024 Jun 18;14(8):jkae136. doi: 10.1093/g3journal/jkae136 (PMC11304950; doi:10.1093/g3journal/jkae136)
Supplement: jkae136_Supplementary_Data [file jkae136_supplementary_data.pdf]

Supplementary Data

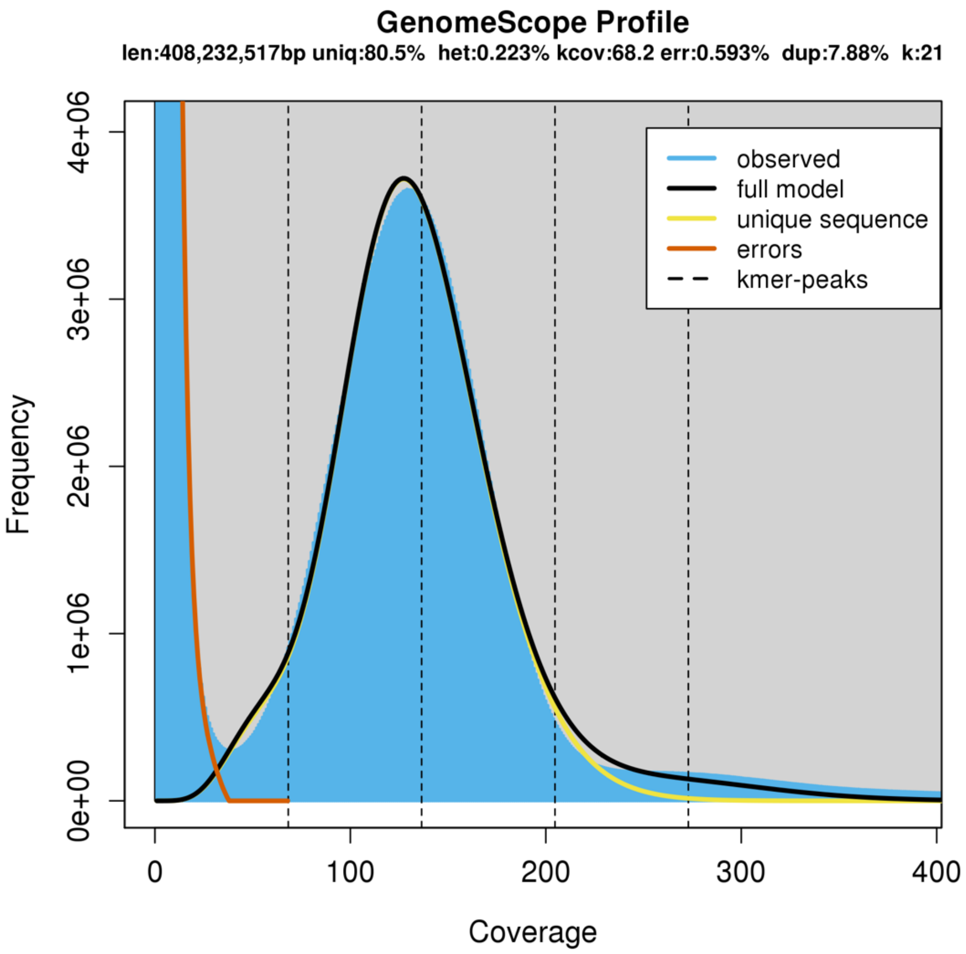

Supplementary Fig. 1. k-mer analysis used to estimate *V. floribundum* genome size and heterozygosity and visualized in GenomeScope. K-mer size was set at 21.

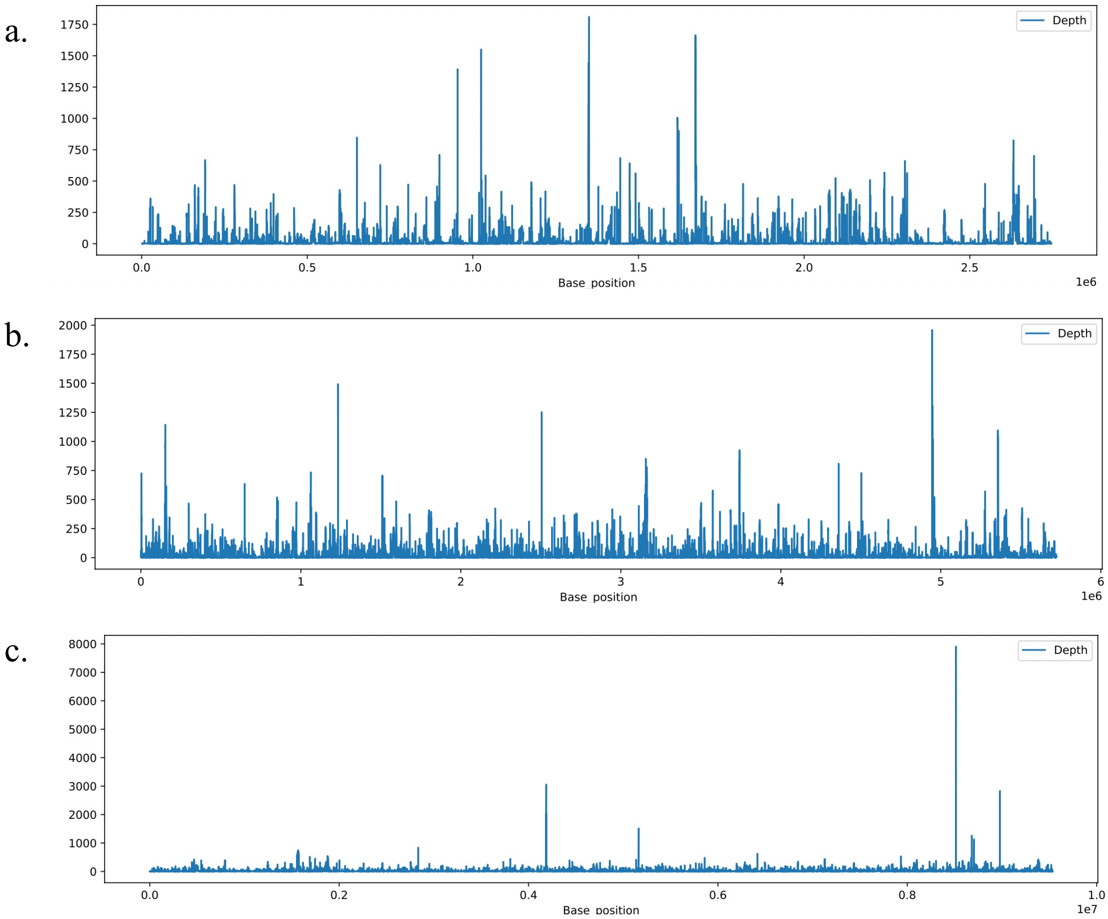

Supplementary Fig. 2. Depth coverage graph of *V. floribundum* of the largest contig using ONT raw reads of **a.** SMARTdenovo assembly, **b.** MaSuRCA assembly, and **c.** Flye assembly.

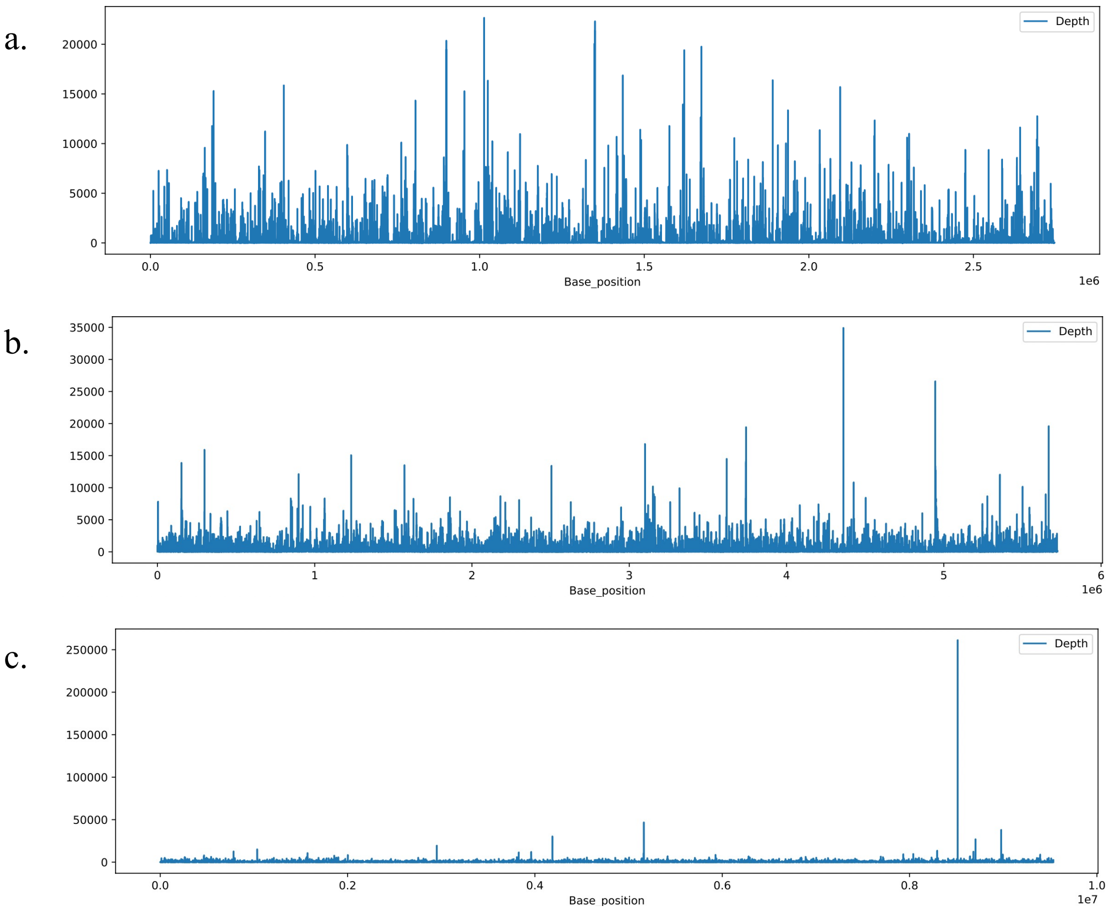

Supplementary Fig. 3. Depth coverage graph of *V. floribundum* of the largest contig using Illumina raw reads of **a.** SMARTdenovo assembly, **b.** MaSuRCA assembly, and **c.** Flye assembly.
